# Stevens-Johnson Syndrome Caused by Enzalutamide: A Case Report and Literature Review

**DOI:** 10.3389/fonc.2021.736975

**Published:** 2021-11-17

**Authors:** Min Deng, Huirong Chai, Meng Yang, Xueman Wei, Wenjun Zhang, Xuebin Wang, Juanjuan Li, Zhuo Wang, Haitao Chen

**Affiliations:** ^1^ College of life sciences and Biopharmaceuticals, Shenyang Pharmaceutical University, Shenyang, China; ^2^ Department of Pharmacy, Shanghai Changhai Hospital, Naval Medical University, Shanghai, China; ^3^ Department of Geriatrics, Shanghai Changhai Hospital, Naval Medical University, Shanghai, China

**Keywords:** enzalutamide, Stevens-Johnson syndrome, drug eruption, cutaneous adverse events, prostate cancer

## Abstract

**Objective:**

Enzalutamide is the most frequently prescribed compound for treating metastatic castration-resistant prostate cancer (mCRPC). Common adverse drug events of enzalutamide are febrile neutropenia, hot flashes, hypertension, and fatigue.

**Methods:**

We present a case of a patient with mCRPC who received enzalutamide and developed Stevens-Johnson syndrome (SJS). The culprit drug was confirmed using the Naranjo Adverse Drug Reaction Probability Scale. Clinical characteristics and management principles were analyzed in combination with literature reports.

**Results:**

SJS occurred within two weeks of enzalutamide therapy. Supportive care such as steroid treatment led to a complete resolution of skin lesions and improved clinical symptoms after three weeks.

**Conclusion:**

Most cutaneous adverse events occur early during enzalutamide therapy, and close observation should be given within two weeks of starting treatment.

## Introduction

Stevens-Johnson syndrome (SJS) is a rare, potentially life-threatening hypersensitivity reaction involving the skin and mucous membranes (1). In SJS, the early rash eventually progresses to the sloughing of large areas of epidermal tissue. Eruption of the cutaneous lesions is preceded by several days of a prodromal phase, comprising of flu-like symptoms (2). Medications associated with SJS have included allopurinol, barbiturates, anti-epileptics, nonsteroidal anti-inflammatory drugs, cephalosporins, penicillins, and sulfonamides (2).

Enzalutamide, a novel androgen receptor (AR) inhibitor, has been approved for the treatment of metastatic castration-resistant prostate cancer (mCRPC) in many countries (3). The binding of enzalutamide to the AR prevents the nuclear translocation of the receptor, thus inhibiting the interaction between AR and DNA (4). The most reported adverse drug reactions (ADRs) are febrile neutropenia, hot flashes, hypertension, and fatigue. However, rare cutaneous adverse events can occur (5). We report a patient who developed SJS after taking enzalutamide for mCRPC treatment for the first time. The characteristics of the skin reactions are described, and the potential mechanisms of the adverse reaction are discussed. The case report contributes to the knowledge base and raises awareness of this rare and potentially life-threatening skin adverse reaction of enzalutamide.

## Case Presentation

In October 2020, a 92-year-old male with prostate cancer was admitted to our hospital. The patient was diagnosed with prostate cancer in July 2014 with a prostate-specific antigen (PSA) level of 30.875 ng/mL. Abdominal ultrasonography showed prostatic hyperplasia, and imaging examination revealed inguinal lymph node metastasis. The patient declined surgical treatment and opted for therapy with bicalutamide and goserelin upon a cancer diagnosis. His PSA level dropped to 1.190 ng/mL after one month of treatment. In June 2017, he was treated with second-line therapy with flutamide due to an increased PSA level. Unfortunately, his PSA level had a rapid doubling rise since June 2018, and the PSA level increased to a maximum of 28.153 ng/mL. In April 2019, he was advised to use abiraterone combined with prednisone as third-line therapy, and this had led to a markedly decreased PSA level (5.070 ng/mL) after seven months.

**Figure 1:**
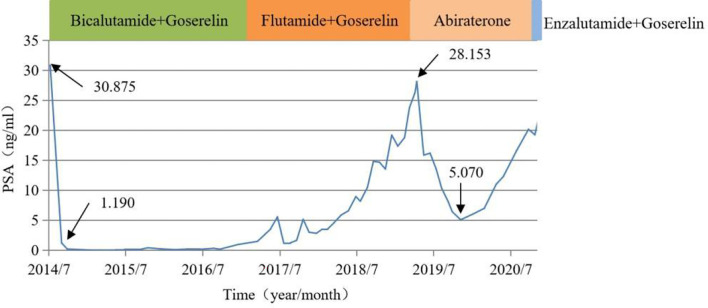
Changes in PSA levels and medications after diagnosis.

**Figure 2:**
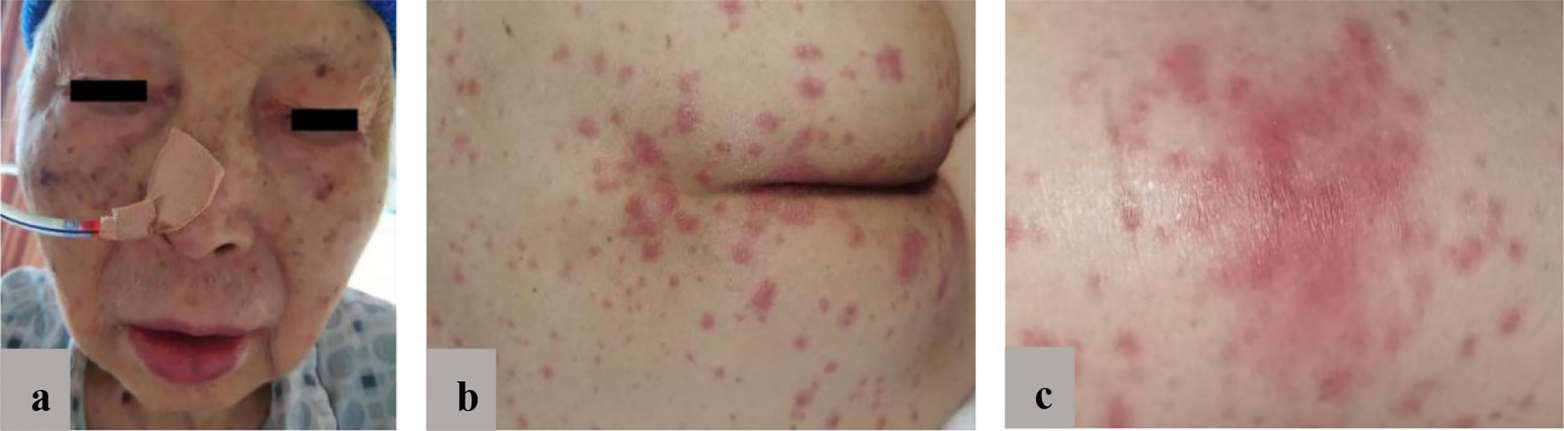
**(A)** Redness and swelling around the eyes; **(B)** Diffuse erythematous plaques involving perianal area; **(C)** Close-up view of the topical eruption.

**Figure 3:**
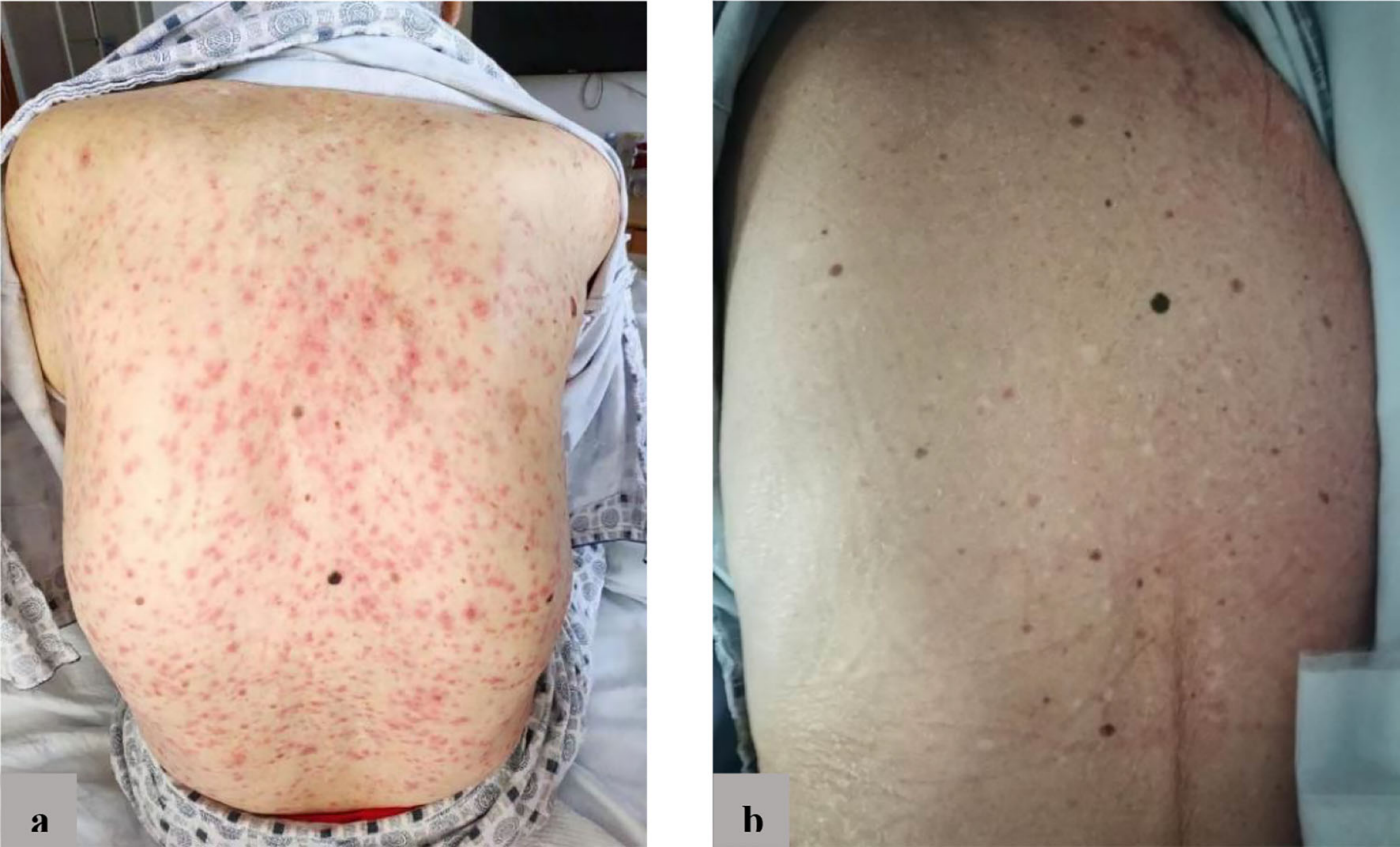
**(A)** Before treatment: diffuse erythema on the back; **(B)** After treatment: erythema basically disappeared.

At the October 2020 admission, the patient’s PSA level was elevated again due to disease progression (**Figure 1**). Subsequently, he received enzalutamide (160 mg by mouth daily) coupled with a subcutaneous implant of goserelin (10.8 mg every four weeks). Ten days after starting therapy, a physical examination found erythematous macules on his perineum, redness and swelling around the eyes, as well as facial edema (**Figure 2**). The patient had no history of dermatologic diseases and was not on any other drugs related to adverse skin conditions. Enzalutamide was not stopped as it was the only effective drug for the mCRPC in this patient, and it was not suspected to cause the patient’s skin condition at that time. Goserelin subcutaneous implant could not be removed due to progressive disintegration over the past four days. Two days later, the lesions evolved to diffuse erythematous plaques all over the body (**Figure 3A**). Moreover, the canthus, lower lip, and perianal area presented with erosion and appeared scabby.

The patient’s temperature fluctuated between 38°C and 39°C. Laboratory findings indicated inflammatory syndrome with serum procalcitonin 0.304 ng/mL, C-reactive protein 90.1mg/L, white blood cell count 9.19×10^9^/L, and neutrophils 76.5%. Based on the clinical manifestations and the time of ADR occurrence, a diagnosis of SJS was made. The likely cause of SJS was suspected to be induced by enzalutamide, and enzalutamide was discontinued. Furthermore, supportive treatment was started, including intravenous hydrocortisone and oral ebastine. After 12 days of stopping enzalutamide, systemic erythema significantly alleviated, and the skin surface appeared desquamated (**Figure 3B**). After three weeks of stopping enzalutamide, the skin lesions completely subsided.

## Discussion

The US FDA approved enzalutamide as an agent to treat mCRPC in the post-docetaxel setting in 2012, and eventually approved enzalutamide as a first-line therapy to manage mCPRC patients in 2014 (6). Enzalutamide has a higher binding affinity to the AR than the first-generation AR antagonists (such as flutamide or bicalutamide). Enzalutamide can also inhibit the intra- or intermolecular N-C interactions, which prevents the translocation of the receptor to the nucleus and therefore blocks subsequent signaling activation (7). Despite the initial response of most prostate cancer patients to androgen deprivation therapy (ADT), the disease typically progresses to a castration-resistant state within 18-24 months (8, 9). In this case, the patient was started with enzalutamide because of the disease progression after ADT. The patient experienced an acute skin reaction after ten days of treatment with enzalutamide. One clinical study of enzalutamide reported that rash occurred in 2.6% of recipients compared to 1.6% in the placebo group, and there were no cutaneous adverse events above grade 3 in both groups (10).

Goserelin is slowly released from the fourth day after subcutaneous implantation and provides stable plasma levels within 12 weeks (11). In this case, the patient had no cutaneous adverse events when taking goserelin alone, and resolution of the skin eruptions occurred without goserelin removal. Thus, enzalutamide was thought to be the culprit of this cutaneous adverse event. According to the Naranjo Adverse Drug Reaction Probability Scale (12), it is highly probable that the SJS was induced by enzalutamide, as the Naranjo probability score was calculated to be five. The clinical manifestation and time to onset of the eruption were consistent with SJS diagnosis caused by enzalutamide. The SJS was assessed to be grade 3 by CTCAE v5.0. The literature recommends complete cessation of the inducing drug, and immunoglobulin or corticosteroid is recommended to treat SJS along with supportive and symptomatic management (13–15). Although there was a possibility that SJS could be related to the enzalutamide dose, enzalutamide was discontinued immediately, considering SJS is a serious skin reaction and the patient’s age. Intravenous hydrocortisone and oral ebastine were given to this patient to manage the SJS, and the patient recovered after three weeks.

We could only find two other cases of cutaneous adverse events to androgen receptor inhibitors in the English literature (**Table 1**) (16, 17). These two cases reported that skin lesions were noticed within two weeks after starting enzalutamide and were improved after hormone therapy. The time of occurrence of cutaneous adverse events and the course of treatment were similar to our case, suggesting that cutaneous adverse events may occur during the early treatment period. The mechanism of enzalutamide-induced cutaneous adverse events is unknown, but it may be mediated by immunological mechanisms (16). One published case report of enzalutamide-induced thrombocytopenia observed a significant platelet count decline two weeks after starting enzalutamide, which recovered 30 days after enzalutamide withdrawal (18). Similarly, adverse reactions occurred about two weeks after the induction of enzalutamide therapy, suggesting that patients should be monitored with clinical symptom checks and blood tests within two weeks after starting enzalutamide therapy. Because of enzalutamide’s long half-life (5.8 days), the eruptions may take one month to resolve even after immediate drug withdrawal and starting symptomatic treatment.

**Table 1:** Case report of cutaneous adverse events caused by enzalutamide.

References	Country	Age (years)	Time of adverse reaction	Therapy
(16)	Japan	87	14 days	Betamethasone
(17)	France	62	10 days	Antihistamine, Betamethasone, Prednisolone (40mg/day)
Our case	China	92	10 days	Ebastine, Hydrocortisone (200mg/day)

In patients with severe ADRs, the drug should be discontinued immediately and changed to an alternative drug therapy. That being said, when the drug is an essential therapy, re-challenging or desensitization can be considered and attempted after evaluating the severity of ADRs and benefits-risks. In a published case report (16), enzalutamide was reinitiated at a low dose after the patient’s rash improved; however, a maculopapular eruption occurred within two days of therapy. The patient continued to receive enzalutamide treatment along with a topical application of betamethasone butyrate propionate ointment. Desensitization primarily targets cells involved in immediate hypersensitivity reactions (i.e., mast cells and basophils). While desensitization has been established to be safe and effective for IgE-mediated drug hypersensitivity reactions, it is not an option in SJS, severe exfoliative dermatitis, drug reactions with eosinophilia and systemic symptoms (DRESS syndrome), or acute generalized exanthematous pustulosis (AGEP) (19, 20).

In this report, enzalutamide-induced SJS was reported for the first time in a 92-year-old man treated for mCRPC. The potential occurrence of this life-threatening skin reaction calls for medical staff to watch for this serious skin adverse event when using enzalutamide, especially for elderly patients. It is worth noting that we could not rule out the possibility that SJS could be related to the 160 mg enzalutamide dose. Also, we could not obtain the patient consent to perform immunophenotypic analysis of natural killer cells and regulatory T-cells, and a histological examination (skin biopsy) to show the infiltrates of lymphocytes and the apoptotic keratinocytes. Further exploration of these limitations may offer more insight into the mechanisms of enzalutamide-induced SJS.

## Conclusions

Cutaneous adverse events, such as SJS, may occur early during enzalutamide therapy, which warrants close observation and blood analysis within two weeks of treatment.

## Data Availability Statement

The original contributions presented in the study are included in the article/supplementary material. Further inquiries can be directed to the corresponding authors.

## Ethics Statement

Consent was obtained from the patient regarding the case report and the use of images.

## Author Contributions

MD wrote and edited of the manuscript. HuC, MY, XWe, and WZ was involved in the identification, selection and management of patient cases. XWa and JL reviewed and edited the manuscript. WZ and HaC was involved in critical revision of the manuscript for important intellectual content. All authors contributed to the article and approved the submitted version.

## Funding

This work was supported by the China Postdoctoral Science Foundation (Grant No.45898), the “2-3-4” Peaking Program of Changhai Hospital (Grant No.2019YXK031), the Clinical Research Plan of SHDC (SHDC2020CR4072), the Shanghai “Rising Stars of Medical Talent Youth Development Program” (Shanghai Municipal Health Commission Planning Personnel Matters [2019]72) and the National Natural Science Foundation of China (81870520).

## Conflict of Interest

The authors declare that the research was conducted in the absence of any commercial or financial relationships that could be construed as a potential conflict of interest.

## Publisher’s Note

All claims expressed in this article are solely those of the authors and do not necessarily represent those of their affiliated organizations, or those of the publisher, the editors and the reviewers. Any product that may be evaluated in this article, or claim that may be made by its manufacturer, is not guaranteed or endorsed by the publisher.

## References

[1] Khalid N Kwak M Leo A Rüegg S Jick SS Meier CR. The Risk of Stevens-Johnson Syndrome and Toxic Epidermal Necrolysis in New Users of Antiepileptic Drugs. Epilepsia (2017) 58(12):2178–85. doi: 10.1111/epi.13925 29027197

[2] Xu L Zhu Y Yu J Deng M Zhu X. Nursing Care of a Boy Seriously Infected With Steven-Johnson Syndrome After Treatment With Azithromycin: A Case Report and Literature Review. Med (Baltimore) (2018) 97(1):e9112. doi: 10.1097/MD.0000000000009112 PMC5943129 29505509

[3] Scott LJ. Enzalutamide: A Review in Castration-Resistant Prostate Cancer. Drugs (2018) 78(18):1913–24. doi: 10.1007/s40265-018-1029-9 30535926

[4] Tran C Ouk S Clegg NJ Chen Y Watson PA Arora V. Development of a Second-Generation Antiandrogen for Treatment of Advanced Prostate Cancer. Science (2009) 324(5928):787–90. doi: 10.1126/science.1168175 PMC2981508 19359544

[5] Davis ID Martin AJ Stockler MR Begbie S Chi KN Chowdhury S. Enzalutamide With Standard First-Line Therapy in Metastatic Prostate Cancer. N Engl J Med (2019) 381(2):121–31. doi: 10.1056/NEJMoa1903835 31157964

[6] Hong JH. Pharmacokinetic/pharmacodynamic Drug Evaluation of Enzalutamide for Treating Prostate Cancer. Expert Opin Drug Metab Toxicol (2018) 14(3):361–9. doi: 10.1080/17425255.2018.1440288 29431540

[7] Alexandra VA Jingchen C Xiaohong L. Mechanisms and Approaches for Overcoming Enzalutamide Resistance in Prostate Cancer. Front Oncol (2018) 8:180. doi: 10.3389/fonc.2018.00180 29911070 PMC5992404

[8] Antonarakis ES Blackford AL Garrett-Mayer E Eisenberger MA. Survival in Men With Nonmetastatic Prostate Cancer Treated With Hormone Therapy: A Quantitative Systematic Review. J Clin Oncol (2007) 25:4998–5008. doi: 10.1200/JCO.2007.11.1559 17971600 PMC4133788

[9] Armstrong AJ Garrett-Mayer E de Wit R Tannock I Eisenberger M. Prediction of Survival Following First-Line Chemotherapy in Men With Castration-Resistant Metastatic Prostate Cancer. Clin Cancer Res (2010) 16:203–11. doi: 10.1158/1078-0432.CCR-09-2514 20008841

[10] Armstrong AJ Szmulewitz RZ Petrylak DP Holzbeierlein JM Stenzl A. ARCHES: Efficacy of Androgen Deprivation Therapy (ADT) With Enzalutamide (ENZA) or Placebo (PBO) in Metastatic Hormone-Sensitive Prostate Cancer (mHSPC). J Clin Oncol (2019) 37(15_suppl):5048–8. doi: 10.1200/JCO.2019.37.15_suppl.5048 PMC6839905 31329516

[11] Cockshott ID. Clinical Pharmacokinetics of Goserelin. Clin Pharmacokinet (2000) 39:27–48. doi: 10.2165/00003088-200039010-00003 10926349

[12] Naranjo CA Busto U Sellers EM Sandor P Ruiz I Roberts EA. A Method for Estimating the Probability of Adverse Drug Reactions. Clin Pharmacol Ther (1981) 30(2):239–45. doi: 10.1038/clpt.1981.154 7249508

[13] Si X He C Zhang L Liu X Li Y Wang H. Management of Immune Checkpoint Inhibitor Related Dermatologic Adverse Events. Thorac Cancer (2020) 11(2):488–92. doi: 10.1111/1759-7714.13275 PMC6997012 31814310

[14] Owczarek W Slowińska M Lesiak A Ciążyńska M Maciąg A Paluchowska E. The Incidence and Management of Cutaneous Adverse Events of the Epidermal Growth Factor Receptor Inhibitors. Postepy Dermatol Alergol (2017) 34(5):418–28. doi: 10.5114/ada.2017.71106 PMC5831275 29507555

[15] Dasanu CA. Late-Onset Stevens-Johnson Syndrome Due to Nivolumab Use for Hepatocellular Carcinoma. J Oncol Pharm Pract (2019) 25(8):2052–5. doi: 10.1177/1078155219830166 30782092

[16] Saito-Sasaki N Sawada Y Okada E Nakamura M. Drug Eruption Caused by Enzalutamide: A Case and Literature Review of Androgen Receptor Inhibitor-Related Drug Eruptions. Australas J Dermatol (2018) 59(2):e133–4. doi: 10.1111/ajd.12694 29878315

[17] Alberto C Konstantinou MP Martinage C Casassa E Tournier E Bagheri H. Enzalutamide Induced Acute Generalized Exanthematous Pustulosis. Dermatol Case Rep (2016) 10(2):35–8. doi: 10.3315/jdcr.2016.1226 PMC5124370 27900064

[18] Murata M Takizawa I Maruyama R Kasahara T Hara N Tomita Y. Enzalutamide-Induced Severe Thrombocytopenia Complicated by a Seizure in a 76-Year-Old Man With Castration-Resistant Prostate Cancer. IJU Case Rep (2018) 2(1):9–11. doi: 10.1002/iju5.12025 32743361 PMC7292079

[19] Hong DIC. Desensitization for Allergic Reactions to Chemotherapy. Yonsei Med J (2019) 60(2):119–25. doi: 10.3349/ymj.2019.60.2.119 PMC6342709 30666832

[20] de las Vecillas Sánchez L Alenazy LA Garcia-Neuer M Castells MC. Drug Hypersensitivity and Desensitizations: Mechanisms and New Approaches. Int J Mol Sci (2017) 18(6):1316. doi: 10.3390/ijms18061316 PMC5486137 28632196

